# Health Impact Assessment of Urban Waterway Decisions

**DOI:** 10.3390/ijerph120100300

**Published:** 2014-12-25

**Authors:** Katrina Smith Korfmacher, Katia Aviles, B.J. Cummings, William Daniell, Jared Erdmann, Valerie Garrison

**Affiliations:** 1 Department of Environmental Medicine, University of Rochester Medical Center, 601 Elmwood Avenue, Box EHSC, Rochester, NY 14642, USA; 2 Corporación del Proyecto ENLACE del Caño Martín Peña, P.O. Box 41308, San Juan, 00940-1308, Puerto Rico; E-Mail: kaviles@martinpena.org; 3 Duwamish River Cleanup Coalition/TAG, 210 South Hudson St, Suite 332, Seattle, WA 98134, USA; E-Mail: bj@duwamishcleanup.org; 4 Department of Environmental and Occupational Health Sciences University of Washington School of Public Health, Box 357234, Seattle, WA 98195, USA; E-Mail: bdaniell@uw.edu; 5 Minneapolis Health Department, 250 South 4th Street, Rm. 510, Minneapolis, MN 55415, USA; E-Mail: jared.erdmann@minneapolismn.gov; 6 University of Rochester Medical Center, 601 Elmwood Avenue, Box EHSC, Rochester, NY 14642, USA; E-Mail: Valerie_Garrison@urmc.rochester.edu

**Keywords:** health impact assessment, urban development, urban health, environment and public health, water quality, recreation, rivers

## Abstract

Health impact assessments (HIA) promote the consideration of health in a wide range of public decisions. Although each HIA is different, common pathways, evidence bases, and strategies for community engagement tend to emerge in certain sectors, such as urban redevelopment, natural resource extraction, or transportation planning. To date, a limited number of HIAs have been conducted on decisions affecting water resources and waterfronts. This review presents four recent HIAs of water-related decisions in the United States and Puerto Rico. Although the four cases are topically and geographically diverse, several common themes emerged from the consideration of health in water-related decisions. Water resource decisions are characterized by multiple competing uses, inter-institutional and inter-jurisdictional complexity, scientific uncertainty, long time scales for environmental change, diverse cultural and historical human values, and tradeoffs between private use and public access. These four case studies reveal challenges and opportunities of examining waterfront decisions through a “health lens”. This review analyzes these cases, common themes, and lessons learned for the future practice of HIA in the waterfront zone and beyond.

## 1. Introduction

Humans use rivers, lakes, and oceans in diverse ways. Waterways provide resources for drinking, transportation, and access to fisheries that have attracted human societies since the earliest times. Cities developed around water bodies that offered access to transportation and trade. Industries often make use of surface waters for water supply, cooling waters, or waste disposal. The recreational and scenic values of waterways also create multiple—and often conflicting—demands on waterfront land and natural resources. These multiple uses have complex implications for human health.

As Bunch *et al*. (2014) point out, “human health and well-being are fundamentally dependent on the governance and management of land and water for sustainability [1].” The impacts of waterways on human health range from very direct (e.g., ingesting water contaminated with bacteria may lead to disease) to indirect (e.g., polluted waterways may discourage physical activity on or near the water [2,3,4,5,6,7,8]. Horwitz and Finlayson (2011) characterize the impacts of wetlands, defined broadly to include waterways, on health in terms of “core human requirements” (provision of food and water), “personal exposure and risks” (psychosocial stressors, water-borne and vector-borne diseases, toxics), and “social determinants” (lifestyles and livelihoods) [9]. Compounding this complexity, changes in a waterway may have both positive and negative health impacts. For example, increasing wetlands may reduce downstream flooding, but some wetlands may also increase human exposure to mosquitoes that carry diseases [9,10,11,12,13]. Although existing policies governing surface water, drinking water, and waterfront land were developed to protect human health and well-being, none provide for comprehensive assessment of the effects of waterway decisions on public health. Health Impact Assessment (HIA) is a promising approach to systematically consider public health in the wide range of decisions, plans, and projects that shape human use of waterways.

Health Impact Assessment (HIA) is a policy and planning tool that supports integration of health into non-health polices by providing information about how proposed actions may affect community health. The HIA framework is a systematic, voluntary, and participatory process. Most HIAs involve reviewing, analyzing and applying multiple sources of information to inform non-health related policies and decisions [14,15,16,17,18]. The role of HIA in decisions varies; Harris-Roxas and Harris (2010) characterize the different types of HIA practice as mandated, decision-support, advocacy, and community-led [19]. Although HIA has been practiced extensively in Europe, Australia, and other countries, only recently has it gained traction in the United States (U.S.) [15]. Several non-profit organizations, government agencies, and foundations have actively promoted the expansion of HIA in varied policy sectors and geographical regions. HIAs in the U.S. have addressed a wide range of decision types, including urban redevelopment, transportation, labor policy, housing inspections, and food policy [20,21,22].

Although each HIA is unique, common evidence bases, analytical techniques, pathways, key health determinants, and community engagement strategies tend to emerge in certain sectors. For example, HIAs of transportation decisions often focus on health determinants such as air quality, noise, and walkability, and on health outcomes such as asthma, obesity, and heart disease. Environmental determinants of health—particularly air quality and the “walkability” of communities—figure prominently in many HIAs. However, few HIAs to date have grappled with health determinants related to waterways.

We analyzed and compared four diverse HIAs related to restoration or redevelopment of waterfront areas: the Above the Falls redevelopment project in Minneapolis, Minnesota, the Caño Martín Peña Environmental Restoration Project in Puerto Rico, the Duwamish River Superfund Site cleanup and restoration in Seattle, Washington, and the Local Waterfront Revitalization Program process in Rochester, New York (Healthy Waterways HIA). All four waterway HIAs were supported by the Health Impact Project, a collaboration of the Robert Wood Johnson Foundation and The Pew Charitable Trusts. These HIAs were conducted on urban waterfront decisions in different regions (Minnesota, New York, Puerto Rico, Washington), impacted varied waterways (rivers, lakes, canals, and coastal embayments), engaged diverse communities, and addressed a wide range of decision types by local, state, and federal agencies (Table 1). All four projects assessed health determinants, environmental dynamics, and human communities impacted by proposed changes in a waterway. We conducted a comparative case analysis of these four water-based HIAs in order to explore the following questions:

- Are there common characteristics of waterway HIAs?
- What are the challenges, needs, and potential to improve the future practice of HIA of waterway decisions?
- What can be learned from the experiences of waterway HIAs for the practice of HIA overall?

Through our exploratory analysis, we identified common themes (characteristic, contributions and challenges of these HIAs) and insights that may be translated to HIA of other types of decisions. The analysis is based on the HIA documents and the authors’ knowledge as participant observers (members of each case’s HIA team). Our goal was not to evaluate the “success” of the HIAs reviewed, but rather to distill lessons from these cases to inform future work. Below, we briefly describe the four case studies as a foundation for addressing these questions. We then discuss these common features, challenges, and lessons learned for the future practice of HIA.

**Table 1:** Four waterway Health Impact Assessments.

Title	Above the Falls	Caño Martín Peña	Duwamish River	Healthy Waterways
Location	Minneapolis, MN	San Juan, Puerto Rico	Seattle, WA	Rochester, NY
Date HIA conducted	2012–2013	2013–2014	2012–2013	2012–2013
Decision context	City of Minneapolis’ decision to revise 20 year-old Above the Falls (ATF) master plan for Mississippi Riverfront areas	Puerto Rican legislature’s decision to fund implementation of the Comprehensive Development Plan for the Caño Martín Peña	US EPA’s proposed cleanup plan for the Lower Duwamish Waterway Superfund site	City of Rochester’s Local Waterfront Revitalization Program (LWRP) development process
HIA primary author(s)	Minneapolis Health Department	Mount Sinai’s Icahn School of Medicine	University of Washington; Just Health Action; Duwamish River Cleanup Coalition/ Technical Advisory Group	University of Rochester Department of Environmental Medicine
Stakeholders/ Community engagement	City and Park Board planning staff; State and local legislators; Private business; ATF citizens advisory committee; Local residents; Tourists; Environmental groups	Local residents; Puerto Rico government; EPA and other federal agencies	Local residents; Tribes; Non-Tribal subsistence fishers; Workers in waterfront industries;EPA and local government officials; Potentially responsible parties	City planning and environmental services staff; Waterfront neighborhood groups; HIA learning collaborative; County staff; Non-governmental interest groups
Decisions assessed	Increase quantity of proposed parkland, trails, jobs, and housing units	Dredging to reopen the waterway, infrastructure improvements including creation of a sewer system, and resident relocation	Proposed cleanup actions, institutional controls for post-cleanup residual contamination	Improvements in beach management, waterfront trails, development planning, water-based recreation, and stormwater management
Primary health concerns	Obesity; Mental health; Environmental quality (air, noise, river water quality); Safety and security; Neighborhood cohesion; Employment	Environmental Quality (mosquito habitat, air, water, sediment); Allergens/asthma; Mental health; Neighborhood cohesion; Gentrification; Physical activity	Fish, sediment contamination; diet, nutrition; Tribal rights; Cultural practices; Gentrification; Social capital; empowerment; Employment; Construction phase impacts	Physical activity; Water quality; Health-supportive resources; Physical safety
Impacts/outcomes of HIA	Integrated in ATF master plan; Strengthened community engagement	Integrated clean up guidelines for workers in restoration project	“Duwamish Opportunity Fund;” Angler survey; Map of alternative fishing sites. Final USEPA decision still pending.	Draft LWRP includes health as a goal; Added sub- policies to LWRP based on HIA recommendations
HIA report	Above the Falls Health Impact Assessment: Ensuring health equity in decision-making. ^a^	Health Impact Assessment of the Environmental Restoration of Caño Martín Peña. San Juan, Puerto Rico. ^b^	Health Impact Assessment: Proposed cleanup plan for the Lower Duwamish Waterway Superfund Site ^c^	Healthy Waterways: A Health Impact Assessment of the City of Rochester, New York’s Local Waterfront Revitalization Program ^d^

Notes: ^a^ www.minneapolismn.gov/www/groups/public/@cped/documents/webcontent/wcms1p-101790.pdf; ^b^ www.martinpena.org; ^c^ deohs.washington.edu/hia-duwamish; ^d^ http://www.urmc.rochester.edu/MediaLibraries/URMCMedia/environmental-health-sciences-center/COEC/projects/documents/HealthWaterways-Report.pdf.

## 2. Waterway Health Impact Assessments: Four Cases from Urban Settings

In this section, we briefly describe the decision contexts, scope of issues addressed, key health determinants, community engagement strategies, environmental dynamics, and outcomes of the four waterway HIAs. An overview of these characteristics is provided in Table 1. Comprehensive descriptions of each HIA are available in the cited HIA reports and other published accounts. These brief summaries provide a foundation for the subsequent discussion of the common lessons learned, challenges, and needs for further research.

### 2.1. Above the Falls (Minneapolis, MN)

The Above the Falls area is a 2-mile long stretch of the Mississippi River running through the northern section of Minneapolis. The historically industrial-use waterfront is surrounded by neighborhoods with predominantly low- and middle-income residents of diverse backgrounds. Industrial land along the riverfront, the associated trucking, noise and odors and the lack of trails or recreational destinations make many parts of the riverfront inaccessible to the public. Residents of the neighborhoods west of the river (North Minneapolis) are predominantly African American with a greater proportion of Asian residents than in the city overall. These North Minneapolis neighborhoods are characterized by higher levels of poverty, unemployment, crime, obesity and premature mortality than other areas of the city. The ATF neighborhoods east of the river (Northeast Minneapolis) have predominantly middle-income and white residents but also have concentrations of low-income Latino residents. Northeast Minneapolis has lower levels of poverty, unemployment and crime compared to North Minneapolis. However, both areas have higher rates of obesity compared to other areas of the city.

In 2000, the city adopted a plan for the Above the Falls area that outlined proposed changes for the Upper Mississippi Riverfront in Minneapolis. A key policy issue was the proposed phasing out of heavy industries that line the banks and promoting a transition to light industry and mixed use (including parks, trails, and housing) that would increase nearby residents’ access to the riverfront. Since adoption of that plan, a number of public and private projects have been completed. However, by 2010, nearly 20 years after its first adoption, city decision makers were questioning whether, how quickly, and to what extent the ATF plan would actually be implemented. To the chagrin of citizens and environmental advocacy groups, city decision makers expressed the need to revise the original plan in order for it to remain viable.

To inform decisions during the plan revision process, the Minneapolis Health Department, in collaboration with the Community Planning and Economic Development Department (CPED), the ATF Citizens Advisory Council (CAC), and the Minneapolis Park and Recreation Board (MPRB), conducted a HIA to investigate the health impacts that could result from key land use alternatives that were proposed for inclusion in the revised Above the Falls (ATF) plan. The health department and CPED worked closely with the CAC, which consists of 30 neighborhood, business and environmental group members, and the MPRB to design and implement the HIA. Community engagement involved more than 20 presentations to local community groups, outreach at community events including four public forums, a design *charrette* organized by youth who reside in the area, “River comment cards” that allowed residents to share short messages about health and the river, and a community engagement survey of nearly 400 respondents.

The HIA focused on the potential health impacts of four specific land use alternatives that were considered for inclusion in the revised ATF plan:

1. To add 108 acres of parkland;
2. To extend existing Riverfront biking and walking trails by 4.2 miles;
3. To add over the long term 3000 jobs; and
4. To add over the long term 1000 new housing units.

The health determinants that the HIA could potentially focus on were numerous. The health department worked with the CAC to narrow down the scope of the HIA. Investigation focused on potential impacts on the following health determinants: obesity, mental health, environmental quality (air, noise, and river water quality), safety and security, neighborhood cohesion and livability, and employment. Impact predictions were developed based on literature review, secondary data, the community engagement survey, and qualitative data collected through targeted outreach strategies with youth and Latino and Lao residents. The impact predictions were based on evidence from the literature and primary and secondary data collected during the HIA.

The final report concluded that although the proposed land use changes could improve health among residents near the ATF area, additional actions were needed to maximize the health benefit for all local residents, not just middle- to upper-income residents and tourists. North Minneapolis is among communities with the worst unemployment disparities between African Americans and Caucasians in the nation [23]. The HIA showed that decreasing proposed housing units along the riverfront and increasing employment and job density would benefit local residents. Maximizing the benefits of employment for local residents, many of whom are low-income people of color, however, would necessitate strong partnerships with the private sector to improve job density and abide by local hiring agreements.

The HIA research and engagement supported the addition of parkland and trails to enhance neighborhood cohesion and perceptions of security and safety. However, additional actions would be essential to extend the benefit of these amenities into surrounding neighborhoods, some of which are perceived to be unsafe due to drugs, crime, and trucking routes along city streets. Through collaboration with local businesses and industries, public transit, urban planners and police, safety and security along the streets that lead to the river could be maximized. Paying particular attention to accessibility for youth, senior citizens, and people with disabilities, as well as ongoing engagement with diverse communities in the process would be essential. A major contribution of the HIA was to increase the ATF plan’s focus on equity and the distribution of benefits of future development.

The Mississippi River is a sacred and recreational destination for people in Minnesota and beyond. However, people in North and Northeast Minneapolis, one of the most racially and culturally diverse areas of Minnesota, have limited accessibility to the Mississippi River. Despite their proximity to the river, many residents currently prefer to go elsewhere for swimming, boating, fishing or other recreational opportunities. However, many residents desire to improve the riverfront in the ATF area as an amenity to be enjoyed by all. According to the HIA Community Input Survey, perceptions of poor water quality were a factor in residents’ decisions to avoid the Mississippi riverfront in the ATF area. The HIA highlighted key issues related to water quality, but noted that many major sources of river pollution are located outside of Minneapolis and that influencing water quality-related decisions within the timeframe of the project was unlikely. In addition, water quality concerns were overshadowed by concerns about poor air quality, loud industrial noises, and traffic congestion. Therefore, although the condition of the waterway was identified as a health concern, recommendations focused instead on future land use along the waterfront.

### 2.2. Caño Martín Peña (Puerto Rico)

The Caño Martín Peña (CMP) is a 3.75 mile long natural tidal channel in San Juan, Puerto Rico that connects the eastern and western portions of the San Juan Bay Estuary, an estuary of national significance and the only tropical estuary in the Environmental Protection Agency’s National Estuary Program. Over 80% of Puerto Rico’s shipped goods pass through the San Juan Bay. Eight low-income communities with over 25,000 people surround the eastern segment of the CMP. These communities suffer from high rates of both acute and chronic disease. The CMP’s flow is severely blocked due to decades of unregulated dumping and construction. Over 3000 residential structures discharge untreated waste into the surface water. Environmental degradation, combined with deficient infrastructure, leads to frequent floods in adjacent neighborhoods during heavy rains, causing unsafe and unsanitary conditions for local residents. As a result of increasingly frequent flood events, residents are exposed to polluted surface water in their homes, open spaces, and roadways.

The need to address the environmental degradation of the waterway, as well as to correct the historical neglect of surrounding communities’ infrastructure, led to the creation of the Comprehensive Development and Land Use Plan for the Caño Martín Peña Special Planning District (the CMP Plan). This involved a government and community planning process spanning two years (between 2002 and 2004) and over 700 community participation meetings.

The resulting CMP Plan focused on environmental restoration and community revitalization through the ENLACE Project. The ENLACE Project is a joint effort between the eight communities surrounding the CMP (Grupo de las Ocho Comunidades aledañas al Caño Martín Peña (G-8, Inc.)), the CMP Community Land Trust, and the quasi-public government agency Corporación del Proyecto ENLACE del Caño Martín Peña.

Despite the CMP Plan’s widespread support from multiple public and private actors, its implementation has been delayed at central government levels due to lack of resources. Meanwhile, environmental conditions continued to deteriorate and impact residents’ health. In 2012, the legislature of Puerto Rico was faced with a decision of whether or not to finance the implementation of the CMP Plan. The HIA was originally intended to inform the Puerto Rican legislature’s funding decision. However, after the Commonwealth’s budget crisis of 2014, the emphasis shifted to a broader audience and mobilizing key entities that would help fill critical shortages in resources and provide ongoing support for the CMP Plan’s implementation. While the CMP Plan involves many aspects including socio-economic development, environmental restoration, and infrastructure components, the HIA focused on three pivotal aspects of the Plan, summarized as the CMP Environmental Restoration Project: (1) dredging of 2.2 miles of the waterway; (2) improvements to the sewer system, storm water drainage, and roadway infrastructure; and (3) demolition of almost 400 residential structures and the relocation of affected residents.

The HIA was conducted by Mt. Sinai’s Icahn School of Medicine in partnership with representatives from the ENLACE Project, government agencies, academic institutions, non-profit advocacy organizations, and community members. The project leadership assembled two groups to provide regular input to this project: a Community Advisory Committee (CAC) consisting of local community residents and a Steering Committee consisting of five public and clinical health professionals familiar with the CMP Plan.

The HIA focused on health determinants ranged from housing, water quality and access to public spaces, to social health determinants including education and social capital. Key health outcomes expected from implementation of the CMP Plan were reductions in gastrointestinal disease prevalence, asthma rates, and dengue infections. Several assessed health issues, including asthma and dermatitis due to high humidity and mold, were found to be related to frequent flooding and stagnant water near homes. Other issues, such as gastrointestinal disease from ingesting contaminated water, focused on direct exposures to poor water quality (bacteriologic and toxic contamination). The HIA also addressed the incidence of mosquito-borne disease (dengue) and bites and stings from displaced wildlife. The CMP Environmental Restoration Project’s potential to improve environmental conditions was linked to increasing physical activity, decreased stress, and reduced exposure to hazards from flood areas and illegal dumpsites. The creation of public spaces for enjoyment and community engagement also was identified as a way to significantly improve health.

As a result of these findings, the HIA recommended full funding and rapid implementation of the CMP Plan, including major infrastructure investments to improve sewage and storm water systems. The HIA also suggested safeguards in order to avoid potential for negative health consequences during the CMP Plan’s implementation. For example, the HIA recommended that construction and dredging worksites should be carefully secured to minimize trespassing to avoid injury, that ecosystem managers should non-toxically prevent incursions of wildlife and mosquitoes into residential areas, and that contractors should construct sound barriers to minimize construction noise. The HIA also focused on strategies to protect the health of and minimize stress experienced by relocated residents, such as providing social workers for relocated families. Another recommendation was to increase support of an existing collective land tenure system called the CMP Community Land Trust (“the Trust”)to help prevent low income residents’ displacement after completion of the restoration project. The Trust has formalized land ownership, which was necessary since many current residents did not have land titles. Under the CMP Community Land Trust, residents individually own their houses, but the land belongs to all members. Increased land value is reinvested in the community for maintenance, guaranteeing improvement in living conditions without increasing cost of living.

In addition to recommendations that would reduce the negative health impacts of current water quality impairments, the HIA addressed the potential future benefits of increased recreational opportunities provided by a restored CMP and publicly accessible waterfront. It highlighted the need for specific programs and actions to maximize the potential health benefits of—and minimize potential harms from—redevelopment for low-income residents of neighboring communities. Finally, as the first HIA conducted in Puerto Rico, the CMP Environmental Restoration HIA raised awareness of the potential of this process to inform other decisions in the Commonwealth.

### 2.3. Duwamish Waterway (Seattle, WA)

The Lower Duwamish River in Seattle has been contaminated by over a century of industrial and urban wastes. As a result, the water, soil, fish and wildlife in this area are polluted with a wide range of toxins, including Polychlorinated Biphenyls (PCBs), carcinogenic Polycyclic Aromatic Hydrocarbons (PAHs), arsenic, and dioxins/furans. The South Park and Georgetown neighborhoods that border this section of the Duwamish River have a high percentage of low-income, non-English speaking, and foreign-born (primarily Asian and Latino) residents. Despite the contamination, the river itself is used for recreation (swimming, boating, fishing), subsistence fishing, and cultural practices. The river is part of the traditional fishing area of the Muckleshoot, Suquamish and Duwamish Tribes.

The USEPA placed this waterway on the Superfund List in 2001. In 2013, the USEPA released its proposed cleanup plan, which includes dredging or capping in place the most highly contaminated sediments, plus enhanced and natural recovery for less contaminated areas. Natural restoration of the shoreline and ecosystem will be conducted separately from the Superfund cleanup plan. Under this proposed plan, contamination would be substantially reduced, but resident fish and shellfish would still be unsafe for subsistence consumption after the 17 year projected active cleanup period. As a result, “institutional controls” (strategies to alter fishing and fish consumption practices) would be required indefinitely, and possibly in perpetuity, to discourage consumption of resident fish or shellfish.

A university and community organization partnership conducted an HIA of USEPA’s plan. The authors included the University of Washington School of Public Health, Just Health Action (a non-profit organization that promotes health equity and community empowerment), and the Duwamish River Cleanup Coalition/Technical Advisory Group (EPA’s Community Advisory Group for the site). Community engagement efforts aimed to elicit the cultural, economic, and health implications of the proposed plan for varied user groups. The HIA was built upon the principles of democracy, equity, sustainability, ethical use of evidence, and a comprehensive approach to promote the health and well-being of all stakeholder groups.

The HIA focused on the impacts of construction, long-term environmental changes, community revitalization, and socioeconomic factors on four different population groups: local residents, affected Tribes, subsistence (non-Tribal) fishers, and workers in local industries. The HIA found that health effects from cleanup-related construction activities should be controllable via best practices, that local jobs will likely be generated by the cleanup efforts, and that environmental restoration could spur community and economic revitalization. However, revitalization could aggravate existing residential gentrification and increase pressures on local industries, with risks of reduced expendable income, displacement of workers, community disruption, and loss of family wage jobs. A key recommendation of the HIA was to convene a Duwamish Valley Revitalization Task Force, with broad stakeholder representation, in order to cumulatively consider the potential impacts on the affected populations and identify opportunities for sustainable and equitable revitalization.

The HIA also examined a variety of impacts related to the contamination of river sediments, fish and shellfish, during and after cleanup. Exposure to contaminated sediment through beach use could be controlled through wash stations at beaches during cleanup, and should be resolved by the cleanup. One of the most significant findings of the HIA, however, was that current and post-cleanup residual contamination of resident fish would pose disproportionate burdens for Tribal and non-Tribal subsistence fishers. The cleanup plan’s reliance on behavior-changing strategies (“Institutional Controls”), such as signage and educational campaigns to discourage eating local fish, could contribute to food and nutritional insecurity for people who value fish in their diet or rely on fishing as a low-cost source of protein, and lead to the substitution of less healthful foods, disruption of social and cultural traditions, and the denial of treaty-guaranteed Tribal rights. The HIA highlighted the complexity of these tradeoffs and the lack of information to assess the overall impacts on health. The HIA recommended a Tribal revitalization fund to help mitigate the negative health effects of continued restrictions to traditional fishing practices.

### 2.4. Healthy Waterways (Rochester, NY)

The City of Rochester’s historical development was closely tied to its water resources. The Genesee River bisects the city, flowing from the southern border where it intersects the Erie Canal to its outlet at the Port of Rochester on Lake Ontario. Today, these waterfront areas contain a mix of low- and middle-income neighborhoods, active and abandoned industrial sites, and parks, beaches, and recreational areas.

The Healthy Waterways HIA assessed Rochester’s Local Waterfront Revitalization Program (LWRP), a state-funded effort to develop a comprehensive plan for the city’s waterfront. Future federal and state actions in the waterfront must be consistent with an approved LWRP, making it a powerful local planning tool. State guidelines for the LWRP include environmental, scenic, and economic impacts, but do not explicitly require consideration of community health.

Healthy Waterways was conducted by staff and faculty in the University of Rochester Department of Environmental Medicine in partnership with the city staff responsible for writing the LWRP and a private planning consultant. Because of the wide range of issues addressed, stakeholder groups were engaged separately on various topics. For example, city and county government staff involved in water quality protection, beach management, and parks advised sections on beaches. Local residents were surveyed and engaged at community meetings around issues related to waterfront housing and commercial redevelopment. Local groups promoting active transportation and cycling were consulted about issues related to trails.

Healthy Waterways focused on potential health impacts of the LWRP through its plans for changes in trails, beach redevelopment, the waterfront built environment, opportunities for water-based recreation, and stormwater management. The HIA projected impacts of these changes on health determinants including physical activity, water quality, health-supportive resources (healthy retail, public spaces, jobs, *etc*.) and physical safety. Key health outcomes included obesity, cardiovascular disease, diabetes, respiratory disease, physical injury (e.g., drowning, accidents, and crime), waterborne disease, and mental health. The HIA report included maps and tables identifying waterfront neighborhoods with disproportionately high rates of disease and poor health conditions.

The Healthy Waterways report included an overview of literature and experiences with these health determinants in other communities, analyses of relevant local data on existing environmental conditions, health, community preferences, and recommendations for each of the topics addressed. As the LWRP was drafted, many of these recommendations were integrated into the plan as draft “subpolicies.” Others, such as a recommendation for an ongoing trails planning and users group, were outside the scope of the LWRP.

Overall, the LWRPs policies were expected to improve health outcomes. The HIA focused on recommendations to enhance these benefits, particularly for vulnerable populations including low income residents, children, and older adults. For example, the HIA recommended additional access points and signage to increase connections between low-income neighborhoods and waterfront trails. Similarly, the HIA recommended land-based exercise resources and water spray playgrounds at beachfront parks to encourage physical activity when the beaches were closed to swimming due to poor water quality. The LWRP included “green infrastructure,” including green spaces to help filter stormwater and protect water quality. The HIA suggested designing these spaces to also provide recreation opportunities during dry weather.

Although many aspects of the Healthy Waterways HIA were common to urban redevelopment decisions in non-waterfront areas, several aspects of the analysis focused directly on the water resources. First, water quality was linked as a health determinant through swimming and boating, where people may be exposed to bacterial pollution. Second, developing access to the river was viewed in some communities as having the potential to increase the risk of accidental drownings. Third, although it was not assessed as a key health determinant, fishing was identified both as a healthful physical and social activity and a potential risk to anglers who consume contaminated fish.

**Community Comments on What Waterways Mean for Health**

- “The Mississippi is a pride of the nation, a basis for folklore, a reminder of the power of water and nature in the midst of urban sprawl.”—Minneapolis resident
- “Well, this is my happiness.”—Caño Martín Peña resident on why she lives there despite the flooding and health conditions
- “It’s (local fish) our spiritual food so it feeds our soul; so it might poison our body, but then we’d rather nourish our soul.”—Duwamish Tribal member
- “I’d rather have fish out of Lake Ontario than store bought, because I know where it comes from.”—Rochester angler

## 3. Case Study Review and Discussion 

These four HIAs differed in terms of geography, populations affected, scope, and key health issues. Although each addressed a decision that involved a waterway, the role of water resources as a health determinant varied from central to ancillary. Nonetheless, they shared several characteristics related to the role of water resources in the decisions they assessed. Below, we highlight six such common features of waterfront HIAs. We then discuss the challenges and needs for the future practice of HIA in waterway decisions, and the lessons that may be learned from these waterway HIAs as they relate to HIAs of other types of decisions.

### 3.1. Common Characteristics of Water-Based HIAs

The four HIAs reviewed shared several common characteristics related directly or indirectly to the role of the waterway in the decision assessed. These include:

1. Water quality may be a health determinant, but the decision being assessed may not encompass the determinants of water quality.
2. The pace of ecosystem health improvements may not match the community’s timeline for human health impacts of the decision.
3. Waterfront land is a limited resource that can limit access to the health benefits of public waterways.
4. Health impacts of waterways are often modified by users’ behaviors and perceptions.
5. Anticipating technical needs, expertise, and assessment approaches is particularly difficult due to the multidisciplinary nature of waterway management.
6. HIA highlights the equity implications of changing waterway uses in ways that existing decision frameworks may not.

Each of these features of waterway HIAs is discussed briefly below using examples from the four cases. Implications for future waterway HIAs are briefly noted and discussed further in the next section. Where relevant, we highlight how water quality is similar to and different from other commonly assessed health determinants, such as air quality.

1. Water quality may be a health determinant, but the decision being assessed may not encompass the determinants of water quality.
  Surface water quality is affected by runoff from and pollutants discharged throughout the entire watershed. Because the decisions addressed by these four HIAs did not encompass the full watershed, the alternatives assessed had limited ability to significantly impact surface water quality. For example, the Genesee River drains an area over 2500 square miles, extending south into rural New York and Pennsylvania. Because most of the river’s nutrient burden runs off of farms throughout this rural watershed, even the strongest storm water controls in the City of Rochester are unlikely to end the algal blooms that plague Rochester’s beaches at the mouth of the river. In Minneapolis, perceived poor water quality is a factor in the lack of recreational use of the ATF riverfront, and additional riverfront parkland (which could improve water quality by mitigating runoff) was being proposed [24]. However, improving water quality as a health determinant was not a major focus of the HIA. In contrast, the Caño Martín Peña plan to treat sewage and restore natural flushing of the estuary was expected to significantly improve surface water quality, although resident fish would still be contaminated from regional pollution. These waterway HIAs all had to contend with the fact that not all stakeholders whose actions affect water quality were part of the decision they were assessing. One solution might be to engage stakeholders in the HIA who were not part of the geographic scope of the decisions being assessed in the HIA (e.g., watershed managers, state environmental agencies, *etc*.).
2. The pace of ecosystem health improvements may not match the community’s timeline for human health impacts of the decision.
  Surface water quality may improve rapidly after reducing discharges of biodegradable waste, as in Caño Martín Peña. However, non-degradable pollutants endure in the sediment and may continue to bioaccumulate in the food chain for decades after the source is removed. In Rochester, the Lake Ontario fish advisory is based on the presence of PCBs and other chemicals that were banned in the late 1970s but continue to persist in the environment and contaminate fish, making them unsafe to eat. Similarly, fish in the Duwamish and the CMP and its adjacent waterways will continue to be unsafe for human consumption even after the proposed cleanup plans are implemented. Thus, the timescale of ecosystem recovery may be much longer than that of the decision being assessed and associated community health interests. In contrast to such long time frames for cleanup of non-biodegradable surface water pollutants, air quality improvements are typically seen soon after pollution sources are reduced. Practitioners and stakeholders should clarify likely time scales for impacts on varied health determinants.
3. Waterfront land is a limited resource that can limit access to the health benefits of public waterways.
  Waterfront land is uniquely valuable for both private and public uses, particularly in densely populated urban areas, and its loss many not be readily replaced. Under the legal tradition of the Public Trust Doctrine, navigable waters in the US are generally considered to be public resources, although its application to waterfront access, priority for water-dependent uses, and use of submerged lands varies by state [25,26,27,28]. Whether privately or publicly owned, waterfront land use controls physical and visual access to the waterway’s public resources. For example, private development of open space in landlocked neighborhoods may be mitigated by building a park in an adjacent area. However, loss of a traditional fishing access point cannot be readily replaced. Only in unusual circumstances, such as the rerouting and uncovering of the Providence River in Rhode Island in the 1990s, are new waterways created [29]. Therefore, when developments displace existing waterway uses, the opportunities to mitigate these losses—and associated health impacts—is limited. In Minneapolis, industrial facilities currently limit residents’ access to the riverfront. The ATF HIA influenced the decision to reduce the proposed number of new private housing developments which would have further limited riverfront access in the ATF area. Instead, the HIA recommended increasing public parks and trails.
  Compounding these threats to public access and traditional uses is that fact that when waterways are improved—for example, by cleaning up a polluted river so swimming is possible—these improvements increase the value of waterfront land for both private and public uses. Preserving public access and other land uses of lower economic value can be challenging in the face of opportunities for profitable private development. The four HIAs discussed the challenges of maintaining visual and physical access to waterways for diverse users and public health benefits that may compete with strong private economic interests.
4. Health impacts of waterways are often modified by users’ behaviors and perceptions.
  Air and surface water are both environmental resources that affect human health, but they differ fundamentally from each other as health determinants. Because everyone breathes air, changes in air quality affect everyone’s health—although children, older adults, and those with respiratory disease may be more vulnerable to pollution. In contrast, the impacts of water quality on health are often modified by users’ behaviors and perceptions. In other words, *water quality* is not a health determinant; rather, *exposure to polluted water through waterways use* is.
  For example, contaminated fish only pose a health risk to those who consume them regularly—generally local anglers. Concerns about drowning risks associated with increased waterfront use in Rochester focused on the vulnerability of people who cannot swim, but who currently do not use the waterfront because of limited accessibility and perceived poor water quality. The contaminated waters of the CMP affect all surrounding residents, but primarily those who come in contact with the flood waters by choice (e.g., swimming) or necessity (e.g., needing to travel during floods). The Healthy Waterways and ATF HIAs noted that clean-up efforts could *increase* health risks from bacteriologic contamination as more people feel comfortable swimming and boating and, as a result, people are more likely to accidentally ingest surface water that is perceived as cleaner but still contains harmful bacteria. Thus, information, knowledge, and beliefs may affect waterway users’ behaviors. Misperception of actual water quality conditions in either direction can negatively affect health: overestimating the cleanliness of water can result in unintentional exposure to pollution; overestimating pollution can unnecessarily limit use of a free resource for exercise, recreation, or fishing. Because of such interactions, it is difficult to predict the combined impacts of the assessed decisions on the water resource and users’ behaviors and perceptions. In response to this dilemma, two of the waterway HIAs (Duwamish and Rochester) emphasized the need to monitor users’ behaviors, knowledge, and perceptions related to the waterway over time.
5. Anticipating technical needs, expertise, and assessment approaches is particularly difficult due to the multidisciplinary nature of waterway management.
  The HIA process emphasizes the importance of scoping health determinants before starting the assessment. Engaging stakeholders in deciding on the key issues, pathways, and outcomes as the process evolves is critical. This creates a fundamental challenge for many HIA teams—how to plan for and access the needed technical skills prior to finalizing the scope of the HIA. Because of the multidisciplinarity, complexity, and uncertainties involved in waterway decisions, this was a challenge to all four HIAs. These HIAs identified significant uncertainties and data gaps that required unanticipated technical resources. For example, the CMP Plan HIA found a lack of data on the incidence of waterborne diseases. This gap existed because the symptoms of waterborne illness (gastrointestinal distress) are often similar to other common illnesses; therefore, cases are seldom reported to local health departments. Even if cases are reported to the health department, it is seldom possible to link them with certainty to exposure to polluted water. Therefore, the CMP Plan HIA team drew upon a community-wide survey and epidemiological study, recently completed by the Ponce School of Medicine, to assess the extent of gastrointestinal disease and its tie in to floodwater exposure As a result of such knowledge gaps, all four waterway HIAs recommended the need for more targeted follow-up studies, additional monitoring, and research to better understand baseline conditions, predict changes, and establish connections between the waterway and human health.
6. HIA highlights the equity implications of changing waterway uses in ways that existing decision frameworks may not.
  A central goal of HIA is to assess the distribution of a decision’s health impacts on diverse populations. However, the multiple uses and values of waterways make these assessments particularly complex. Diverse uses, users, and values are common in waterway decisions; HIA highlights the equity implications of these competing demands. Waterfronts are valued for industrial, transportation, residential, aesthetic, cultural and recreational uses. The decision being assessed generally only encompasses a subset of these uses, making it challenging to predict the overall health impacts of any one decision on various populations. For example, the ATF HIA pointed out that the overall impact of the redevelopment plan on eliminating the unemployment gap between African Americans and Caucasians in North Minneapolis depends on the cooperation of private industries located in the ATF area (e.g., whether or not they hire local workers or increase job density). The ATF plan itself may have little impact on actually securing such commitments from private industry. In the Duwamish, the community resident and industry stakeholder groups have many problems in common, such as the threat of gentrification. However, these same groups tend to be on opposite sides on river cleanup topics, where the community generally wants the best possible cleanup but the potentially responsible parties (PRPs)—including the City and County government—want the most efficient and affordable cleanup. The CMP Plan currently serves as a bridge between the government’s economic priorities and the low-income communities surrounding the CMP. Although the CMP Plan’s implementation will result in the relocation of low-income residents, the guidelines of the ENLACE Project and the Land Trust aim to protect the communities from displacement. The CMP Plan also prioritizes infrastructure improvements in adjacent communities, rarely addressed in ecosystem restoration projects near low-income neighborhoods. As discussed below, such dynamics have significant implications for equity and health disparities over the long-term.

The six characteristics described above are not unique to waterway HIAs, but in each of these cases they were related to the role of water resources in the decision being assessed. Taken together, these characteristics contribute to the complexity of waterway HIAs. They also clarify why health disparities and equity concerns figure so prominently in all four cases. Guidelines for conducting HIAs emphasize the importance of attending to decisions’ impacts on diverse groups of people, particularly vulnerable populations. The waterway HIAs called attention to the distribution of benefits among different population groups in ways that might not otherwise have been considered as part of the normal plan or decision-making process. In each case, the HIA process also highlighted opportunities to lessen (or mitigate increases in) health disparities. Below, we discuss three ways in which HIAs may enhance consideration and reduction of the potential health equity impacts of waterway decisions.

First, as noted above, negative health impacts of polluted water resources are modified by behavioral choices and perceptions. These human factors may affect risks disproportionately among different groups of people. For example, certain populations may be more vulnerable because they do not receive, understand or perceive risks communicated in public health warnings—such as staying out of the water after a heavy rain that washes polluted runoff into the waterway—intended to prevent their exposure to contaminants. Or, they may receive and understand the messages but have limited options to comply. Subsistence fishers who rely on fish as an important source of free protein may be less likely to comply with fish consumption advisories than recreational anglers. As in the case of the Duwamish, the cultural value of the resource (in this case, resident fish) to certain groups may outweigh the perceived risk of chemical contaminants.

Second, because waterfront land is so valuable for housing and commercial development, gentrification is a common byproduct of the redevelopment of formerly industrial and low-income residential waterfronts. Waterfront developers profit more by targeting higher-income sectors of the population. When planning decisions improve property values, low-income residents may no longer be able to afford living in close proximity to the waterway and may lose access to its health-promoting resources, aesthetics, and cultural values. This may provide a public good by increasing the city’s tax base, but can also contribute to displacement of low-income populations currently living in the waterfront zone. Involuntary relocation may contribute to stress and negatively impact health. Thus, statistical health indicators (such as life expectancy, obesity, or asthma rates) for the waterfront neighborhoods may improve as wealthier and healthier people move in, whereas the health of the original residents is compromised. All four HIA cases address the potential for revitalization efforts to displace current residents and call for specific actions to monitor and mitigate health impacts on these populations.

Third, although waterfront revitalization is often supported with public funding, most of the related development is private. Unless visual or physical access for the public is required by regulation, revitalization efforts may decrease access. As a result, waterfront decisions that have overall benefits for the environment, economy, and population health may also have the unintended consequence of increasing health disparities. All four HIAs point out that plans to improve the conditions of the waterway may not benefit current, low-income residents and waterfront users who have the greatest health needs (for instance, for access to free exercise resources like walking and biking trails). Compounding this inequity, a waterway may be the only natural environment readily accessible to low income residents, whereas more affluent neighbors can travel to more distant parks, trails, and beaches. For these reasons, the waterway HIAs encouraged planners to incorporate opportunities for recreation, exercise, active transportation, and public access that can contribute to reducing health disparities.

### 3.2. Future Needs for Waterway HIAs

Several common challenges faced in these cases suggest opportunities to enhance the future practice of HIAs of waterway decisions. These include improving metrics for assessing waterway health determinants, improving the evidence base connecting waterways to health, and developing models of community engagement suited to the nature of waterway decisions. Further efforts in these areas could expand the consideration of public health and health equity in future waterway decisions.

#### 3.2.1. Identify “Pathways” by Which Waterways Impact Health

These HIAs struggled with a lack of appropriate data sources and indicators for the health impacts of waterways. Surface water quality standards are meant to protect human health by categorizing surface waters by their suitable uses. State environmental and health departments typically monitor water quality and publicize conditions that impair uses, for example by closing beaches with high levels of bacteria. Similarly, state health departments issue fish consumption advisories based on contamination of specific waterbodies. However, these single-issue policy systems do not reflect the full health values of a waterway. The Duwamish example shows this most strikingly, in that the Tribes’ cultural practices and nutrition are affected by not being able to consume resident fish. Other values not reflected in water quality regulations include the potential of waterbody or waterfront parks as resources for physical activity, or as health-supporting resources that promote stress reduction, social cohesion, community pride, or as aesthetic destinations that can drive tourism. Developing sample pathway diagrams for a range of potential waterway health impacts may help other groups more readily conceptualize whether—and if so how—their water resource impacts health. Compiling an overview of waterway health impacts may also be a useful resource for future HIA practitioners. Such resources may be particularly useful because of the wide range of technical expertise, complex natural systems dynamics, and government programs relevant to waterways with which HIA practitioners may not be familiar.

#### 3.2.2. Establishing an Evidence Base for Waterway Decisions’ Impact on Health

As noted above, environmental and health agencies’ standards for water quality do not fully account for waterway decisions’ impacts on health. Thus, additional tools are needed to help HIA teams predict how waterway decisions may affect health outcomes. This is particularly challenging because of how education, perception, and use patterns may change in ways that modify the waterways’ impact on health. For example, one study found that perceptions of cleaner water led to more contact recreation, exposing more people to bacteria [4]. Such investigations of the cumulative impacts of regulation, education, and environmental quality are rare. In order to develop this evidence base, more comprehensive monitoring and evaluation of the impacts of waterway decisions on behaviors is essential. One example highlighted by the Duwamish HIA was the need for rigorous evaluation of the effectiveness of institutional controls. Because of the behavior—and institution—mediated impacts on health, this ‘evidence base’ should include qualitative case studies as well as quantitative data.

#### 3.2.3. Developing Models of Community Engagement for Waterfront-Based HIAs

Community engagement is a core principle of HIA. Many HIAs convene a stakeholder advisory committee to inform the scope and process of recommendations. Because waterways HIAs tend to involve multiple populations, stakeholders, and user groups, community engagement may be particularly complex for these projects. Each HIA had an overarching advisory group consisting primarily of professionals and organized interest group staff. In addition, each HIA identified several distinct “affected populations” with interests in different aspects of the decision. Different community engagement approaches were implemented with each of these groups. However, each HIA also utilized several mechanisms for obtaining input from affected community members and residents. Guidelines for development of such multi-faceted community engagement programs could be helpful to future waterway HIAs.

### 3.3. What Can the Practice of HIA Learn from Waterways?

We identified three types of recommendations highlighted in these four waterway HIAs that may be relevant to the practice of HIA in non-waterway decisions. These recommendations relate to monitoring changes in waterway users’ behaviors during implementation, actions beyond the scope of the decision being assessed, and needs for institutional changes to sustain positive impacts on public health.

#### 3.3.1. Recommendations for Monitoring the Human Ecosystem

Guidelines for the practice of HIA emphasize evaluating the health effects of the decision [15,18]. One of the key themes identified in these four HIAs was the role of users’ behaviors and perceptions in modifying the health effects of the decision. Accordingly, all of them called for socio-economic monitoring during implementation. For example, water quality may improve, but people will not change their use of the waterway (for swimming, boating, *etc*.) unless they believe the water to be safe. Decision makers often plan to monitor the decision’s impact on the physical system (e.g., water quality, fish contamination, *etc*.). However, it may be up to the HIA team to recommend continued monitoring of the human ecosystem (e.g., beach user surveys, angler surveys, trail and park use monitoring, perceptions of crime and safety, changes in perception/knowledge about environmental conditions among different community groups, *etc*.). Recommending socioeconomic monitoring may be relevant to HIAs of other decisions in which values, perceptions, and behaviors mediate health impacts.

#### 3.3.2. Recommendations beyond the Recommendations

Each of these HIAs offered recommendations to the decision makers responsible for the policy, plan, or funding choice they assessed. However, each also identified a number of key decisions outside the scope of the assessed decision that had a strong bearing on the assessed health outcomes. For example, the ATF plan guided public decisions about the waterfront, but could not control the private industries’ choices. Therefore, the HIA recommended reaching out to industries to encourage them to hire local workers and develop their operations consistent with the waterfront plan. Similarly, Healthy Waterways recommended that beach managers improve communication to the public about the causes, conditions, and consequences of water quality-based beach closures. Public outreach was outside the LWRP’s scope, but the HIA noted that communication is critical to protecting public health during its implementation.

These kinds of “recommendations beyond the recommendations” may be particularly common due to the diverse use, multi-sectoral, and inter-institutional nature of managing waterways. However, it is not uncommon for HIAs in other issue areas to identify health-promoting changes outside the scope of the decision addressed. Anticipating these additional recommendations at the outset might allow the HIA team to garner the resources, structures, and supporters needed to promote their implementation.

#### 3.3.3. Recommendations for Institutional Change

Several of the HIAs noted the need for new institutions to support implementation of the decision in order to maximize health benefits. Coordination is a common challenge in waterfront management; adding health to the mix further complicates inter-institutional issues. For example, the Healthy Waterways HIA recommended formation of a trail users group to coordinate neighborhood groups, private recreational events, and city staff responsible for infrastructure improvements, signage, and maintenance. Similarly, the Duwamish HIA recommended the establishment of a multi-sector Revitalization Task Force to guide investment in and implementation of the HIA recommendations.

Improved institutional coordination was recommended in all four waterway HIAs. This need may not be as clear cut in HIAs with a single decision-making and implementing body. However, since HIA by definition involves bringing a new interest (health) into interaction with non-health institutions, many HIAs may recommend new systems for coordination in order to maximize health benefits. Given the frequency of such recommendations, it might be useful to build support for coordinating mechanisms into the HIA process from the outset.

## 4. Conclusions

Government officials, health care professionals, and communities all recognize the critical role the environment plays in human health. Many water quality protection policies refer to protection of human health as a primary goal. However, single-sector environmental regulations are insufficient to protect public health equitably. Similarly, land use planning efforts are often justified as essential to protection of public health, but lack a framework to assess how changes in the waterfront impact human health. HIA can complement the single-sector approach of existing systems for managing waterways to promote more comprehensive protection of public health.

The four HIAs reviewed here demonstrate the potential contributions of including health in waterway decisions in diverse policy-making, geographic, and community contexts. The commonalities among these cases suggest areas for further development of tools to support waterway HIAs. Lessons learned from these cases may also be useful for HIAs in other decision arenas, particularly those involving environmental determinants of health, diverse communities, competing resource uses, and health impacts that are modified by people’s behaviors, knowledge, or beliefs.
